# Brachial Plexus Injury Associated with Median Sternotomy during Cardiac Surgery: Three Cases of C8 Radiculopathy Due to the Fracture of the First Rib

**DOI:** 10.3390/diagnostics11101896

**Published:** 2021-10-14

**Authors:** Yu Jin Im, Min Soo Kang, Sun Woong Kim, Duk Hyun Sung

**Affiliations:** 1 Department of Physical Medicine and Rehabilitation, Samsung Medical Center, School of Medicine, Sungkyunkwan University, Seoul 06351, Korea; yujin93.im@samsung.com (Y.J.I.); ekangms@naver.com (M.S.K.); 2 Department of Physical Medicine and Rehabilitation, Guro Hospital, Korea University, Seoul 08308, Korea; srdysw88@naver.com

**Keywords:** thoracic surgery, sternotomy, rib fractures, brachial plexus neuropathies, radiculopathy

## Abstract

In cardiac surgery, median sternotomy is often necessary during certain surgical processes and it can cause the rare complication of brachial plexus injury. Retraction of the rib cage during median sternotomy may produce a fracture of the first thoracic rib at the costovertebral junction which might penetrate or irritate the lower root of the brachial plexus. Because the C8 ventral root is located immediately superior to the first thoracic rib, the extraforaminal C8 root is thought to be the key location of brachial plexus injury by the first rib fracture. This report describes three cases of brachial plexus injury after median sternotomy in a single center. In our cases, fracture of the first rib and consequent brachial plexus injury is confirmed with imaging and electrophysiologic studies. The fracture of the first rib is not detected with standard plain images and it is confirmed only with CT or MRI studies. Advanced imaging tools are recommended to assess the first rib fracture when brachial plexus injury is suspected after median sternotomy.

## 1. Introduction

In various cardiac surgeries, median sternotomy is mandatory during the surgical process and the sternal retractor is used to provide sufficient surgical exposure. Brachial plexus injury (BPI) has been reported as a well-known complication in these surgical situations [1,2,3]. Previous reports suggested that retraction of the rib cage during median sternotomy may produce a fracture of the first thoracic posterior rib and the lateral edge of the fractured first rib might penetrate or irritate the lower root of the brachial plexus (BP) [3,4]. Levin et al. stated that sternotomy-related BPI showed predominant damage in the C8 nerve root distribution, suggesting that this lesion is localized at the level of the anterior primary rami of the C8 nerve root, not in the lower trunk of the brachial plexus [5]. However, a fracture of the first rib and subsequent development of the C8 radiculopathy has not been confirmed clearly with the imaging studies as well as the electrophysiologic studies in the subsequent literatures. We report a case series to describe the clinical, electrophysiologic, and radiologic features of C8 radiculopathy due to mechanical injury to the extraforaminal C8 nerve root by the fracture of the first rib after median sternotomy.

## 2. Case Report

### 2.1. Case 1

**Figure 1:**
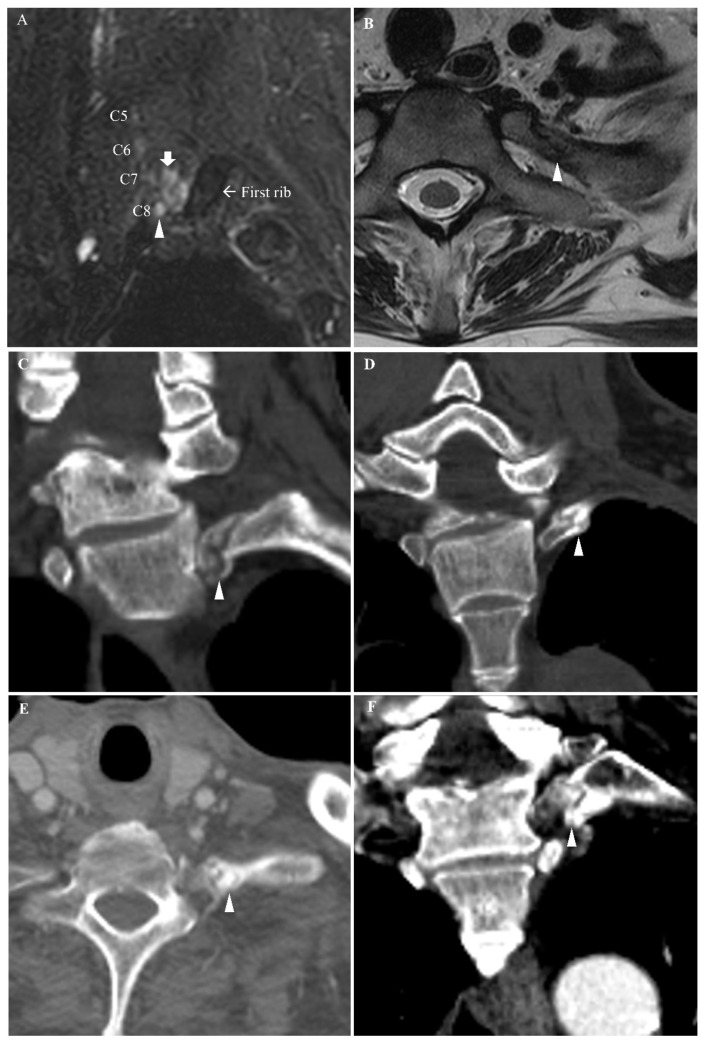
(Case 1: (**A**,**B**)) Brachial plexus and cervical spine MRI findings. (**A**) Parasagittal T2-weighted fat suppression image shows increased T2 signal intensity at left C8 extraforaminal nerve root (arrowhead). T2 Hyperintense area between left C8 root and the left first rib suggests soft tissue edema (short arrow). (**B**) Axial T2-weighted image of the cervical spine shows oblique fracture at the neck of the left first rib between the costovertebral joint and the costotransverse joint without displacement or dislocation (arrowhead). (Case 2: (**C**,**D**)) Coronal chest CT images show (**C**) nondisplaced fractures of the neck of the left first rib (arrowhead) and (**D**) the second rib (arrowhead) without dislocation at the costovertebral and costotransverse joints. (Case 3: (**E**,**F**)) Callus formation was noted at each fracture site. Chest CT images show oblique fracture of the neck of the left first rib and callus formation (arrowhead) in (**E**) axial image and (**F**) coronal image.

A 28-year-old man with underlying dilated cardiomyopathy received heart transplantation through a median sternotomy because of heart failure. When he recovered from the general anesthesia, the patient felt pain and a tingling sensation in his left forearm medial side and 4th–5th digits at an intensive care unit. Two weeks later, when his general medical condition was improved, he recognized the weakness of the left-hand grip and clumsiness of fine motor activities of the left hand, such as pinching. On physical examination at the consultation (four weeks after the heart transplantation) Spurling’s test provoked aggravation of paresthesia in left 4th–5th fingers. Tinel’s sign was positive in percussion to the left lower interscalene space. On a sensory test, there was hypesthesia of pin prick and touch sense at the left forearm medial side and the fifth finger. Motor strength of the left wrist flexor and hand intrinsic muscles was decreased (grade 2~4 on Medical Research Council (MRC) scale). Grip strength of the left hand was 12 kg force (kgf) (33% of right-hand grip strength) and lateral pinch strength of the left hand was uncheckable with the hydraulic pinch gauge. In electrophysiologic studies, sensory nerve action potential (SNAP) amplitude of the left ulnar nerve (24.2 μV) and left radial nerve (14.3 μV) was slightly decreased compared to that of right side (Table 1). In motor NCS, compound muscle action potential (CMAP) amplitudes of the left median, left ulnar and left radial nerves were also reduced by 2.5, 2.7 and 1.3 mV, respectively on distal stimulation (Table 1). Electromyography revealed denervation potentials at the left C8 myotome muscles; however, no denervation potentials were detected at abductor pollicis brevis (APB) and lower cervical paraspinal muscles (Table 2). These findings suggested the left C8 nerve root lesion probably occurred at the extraforaminal nerve root before forming the lower trunk of BP. To figure out the lesion site, he underwent imaging studies for the BP and cervical spine. A T2-weighted image of BP magnetic resonance imaging (MRI) showed a T2 high-signal lesion in the soft tissue between the C8 root and the first rib and increased T2 signal intensity of the left C8 nerve root from the neural foramen to the lower trunk (Figure 1A). T1-weighted MRI showed an oblique fracture of the neck of the left first rib between the costovertebral joint and the costotransverse joint without displacement or dislocation at each joint (Figure 1B). Standard plain radiography of the chest and the cervical spine did not reveal this fracture. On the basis of results of electrophysiologic studies and imaging studies, he was diagnosed with left extraforaminal C8 radiculopathy due to the neck fracture of the left first rib during median sternotomy and was instructed to avoid excessive chest wall or shoulder movement. When followed in an outpatient clinic 3 months after the heart transplantation, the grip strength of his left hand was improved to 16 kgf (62% of right-hand strip strength). At nine months after transplantation, muscle strength of finger flexors and abductors improved to grade 5 in MRC grade. Paresthesia and hypesthesia of left fourth and fifth fingers disappeared.

### 2.2. Case 2

A 46-year-old man received a total aortic arch replacement through a median sternotomy due to infection of the previously grafted aortic arch. On awaking from general anesthesia, he felt a tingling sense of the left fourth and fifth fingers and loss of fine movement of the left hand. On physical examination at one week after the surgery, left forearm and hand muscles showed motor weakness of the finger abductors and extensors (MRC grade 3~4). On sensory examination, pinprick hypesthesia at the area innervated by left ulnar nerve was noted. Sensory and motor NCS conducted at 2 weeks after surgery showed SNAP amplitude of the left ulnar nerve (21.6 μV) was slightly decreased compared to that of the right side (28.1 μV) (Table 1). No definite electrophysiologic abnormality was noted in median and ulnar motor NCS (Table 1). Electromyography revealed denervation potentials at the left ulnar nerve innervated C8 myotome muscles without denervation potentials of the left lower cervical paraspinal muscle (Table 2). The electrodiagnostic examination suggested left C8 radiculopathy at the extraforaminal nerve root or left ulnar neuropathy. A CT scan of the cervicothoracic spine conducted 5 weeks after surgery demonstrated the fracture involving the neck of the left first and second rib without displacement (Figure 1C,D). This fracture was not detected with standard plain radiography of the chest. The C8 nerve root was thought to be injured at the extraforaminal space by the fractured first rib. When followed at outpatient clinic 2 months after the surgery, motor strength of the left intrinsic hand muscles was recovered to grade 5 in MRC grade. Paresthesia of left fourth and fifth fingers disappeared.

### 2.3. Case 3

A 69-year-old man received cardiac transplantation through a median sternotomy due to heart failure. He complained of numbness of the left fourth and fifth fingers in the intensive care unit when the tracheostomy tube was removed at 2 months after the transplantation. His neurologic symptom was developed after the heart surgery, but he could not explain his discomfort to the medical team because the tracheostomy tube impaired verbal communication with spoken language. On physical examination at 10 weeks after heart transplantation, left finger abductors showed mild motor weakness (MRC grade 4) and Spurling’s test was negative. In electrophysiologic studies conducted at 15 weeks after the surgery, SNAP of the left ulnar nerve was not evoked (Table 1). Motor NCS showed reduced CMAP amplitude of bilateral ulnar nerve (7.8 mV on the right side and 1.7 mV on the left side) (Table 1). Electromyography revealed denervation potentials at rest and reduced recruitment of MUAPs on maximum volition at the left ulnar C8 innervated muscles (Table 2). The chest CT scan demonstrated an oblique fracture of the neck of the left first rib with callus formation, but displacement was not noted (Figure 1E,F). Fracture of the first rib was not evident in standard plain radiography of the chest. The C8 extraforaminal nerve root was thought to be injured by the fractured first rib and surrounding callus. When followed up in outpatient clinic at 7 months after transplantation, he showed left hemiplegia due to right middle cerebral artery territory infarction at 4 months after heart transplantation. Thus, prognosis of his left C8 radiculopathy could not be determined.

## 3. Discussion

In these three cases, a Cooley sternal retractor was used to maintain sufficient opening of the chest cage for the cardiac surgery and neurologic symptoms developed immediately after the return of consciousness after surgery. The electrophysiological examinations revealed the extraforaminal C8 nerve root lesions. CT and MRI studies showed a neck fracture of the first rib, which was not detected with standard plain images of the chest/cervical spine. These findings support the hypothesis that extraforaminal C8 root injury was caused by the first rib fracture that had occurred due to mechanical stress during median sternotomy.

The incidence of BPI during open heart surgery has been reported to be between 2 and 38% and the majority of BPI were lower trunk lesions [6]. A prospective study reported 12% incidence of lower truncal lesions of the brachial plexus confirmed with electrophysiologic studies after open heart surgery [7]. Excessive spreading force on the upper chest cage by a sternal retractor during cardiac surgery can cause fracture of the upper ribs with an incidence of 4 to 16% [8]. Anatomically, the ventral ramus of the C8 extraforaminal nerve root is located immediately anterior and superior to the first thoracic rib [5]. Therefore, lower trunk the BP or C8 extraforaminal nerve root may have direct mechanical injury from displaced fractured first rib during cardiac surgery [5]. An autopsy study demonstrated fractured first rib impaling brachial plexus in 11 of 15 patients [9]. In our cases, superior or anterior displacement of the fracture ends of the first rib were not observed in postoperative imaging studies. Although it is a possible scenario that BP was injured by superior dislocation of the fractured end during retracted period of the chest cage and the fracture might be reduced when the sternal retractor is removed, this scenario seems unlikely because imaging studies of our cases show only fracture line and surrounding callus formation without any displacement of the fractured ends. Considering prompt recovery of motor strength to near normal strength in case 1 and case 2, it is more likely that C8 radiculopathy is caused indirectly by swelling, inflammation, or hematoma at adjacent soft tissue due to the neck fracture of the first rib, rather than direct impalement injury by fracture tips.

Previous studies reported that BPI was more prevalent at left side than right side after cardiac surgery [7]. Greenwald et al. studied 24 patients undergoing heart surgery with postoperative bone scans [10]. Bone scans revealed 14 on the right and 30 on the left fractures. In our study, BPI of all 3 cases was left-sided. Because the chest cage is retracted more widely to left side for securing the surgical field during surgery, the left side seems to be more frequently involved in occurrence of fracture of the first rib and secondary BPI than the right side.

In our cases, we could not detect fracture of the first rib with standard plain images of the chest and cervical spine. It is confirmed only with CT or MRI studies. It is recommended to perform advanced imaging tools to identify the first rib fracture and to assess the BP in the patients with suspected lower trunk lesion of the BP or lower cervical radiculopathy after the median sternotomy.

## 4. Conclusions

In summary, brachial plexus injury is a potential rare complication of the cardiac surgery through median sternotomy. Direct and indirect injury of the C8 extraforaminal nerve root occurred in case of the neck fracture of the first rib, especially at left side, due to retraction of the upper chest cage during median sternotomy. Although its prognosis is relatively good, reduction in opening degree of the sternum and caudal placement of sternal retractor is recommended to prevent this preventable complication after median sternotomy. If C8 radiculopathy or lower trunk lesion of the BP after median sternotomy is suspected, imaging studies such as CT or MRI can be helpful to assess the fracture of the first rib and consequent brachial plexus injury.

## Figures and Tables

**Table 1:** Results of sensory/motor nerve conduction studies.

	Nerve	SNAP Amplitude (µV) (Baseline to Peak)				CMAP Amplitude (mV) (Baseline to Peak)			
		Record	Right	Left *	NLL	Record	Right	Left *	NLL
Case 1	Median	Digit II	37.6	39.4	>28.0	APB	6.7	2.5	>8.3
	Ulnar	Digit V	28.6	24.2	>23.8	ADM	7.6	2.7	>8.0
	Radial	1st DWS	29.6	14.3	>15.0	EIP	3.7	1.3	>2.0
	MABC	Forearm	14.8	13.1	>5.5				
Case 2	Median	Digit II	38.5	35.0	>16.3	APB	9.6	8.8	>7.5
	Ulnar	Digit V	28.1	21.6	>15.0	ADM	11.2	10.2	>7.5
	MABC	Forearm	13.0	14.0	>8.5				
Case 3	Median	Digit II	29.5	31.4	>14.9	APB	6.7	7.5	>5.7
	Ulnar	Digit V	15.4	NR	>14.9	ADM	7.8	1.7	>7.3
	DUCN	4th DWS	12.4	4.4	>8.0				
	MABC	Forearm	12.0	6.7	>5.8				

Asterisk: Affected side is left in all 3 cases; NLL: Normal lower limit; MABC: medial antebrachial cutaneous; DUCN: dorsal ulnar cutaneous nerve; DWS: dorsal web space; APB: abductor pollicis brevis; ADM: abductor digiti minimi; EIP: extensor indicis proprius; NR: no response.

**Table 2:** Results of needle electromyography.

	Muscle (Left)	ASA		MUAP			R
		PSWs	Fibs	Amp	Dur	Phases	IP
Case 1	APB	None	None	↑	↑	Normal	Reduced
	ADM	1+	1+	Normal	↑	Poly	Reduced
	FDI	1+	1+	Normal	Normal	Poly	Reduced
	EDC	None	1+	Normal	↑	Poly	Reduced
	EPL	1+	None	Normal	Normal	Poly	Reduced
	EIP	None	1+	Normal	↑	Normal	Reduced
	Lower cervical PSP	None	None				
Case 2	APB	None	None	Normal	Normal	Normal	Complete
	ADM	1+	None	Normal	Normal	Normal	Reduced
	FDI	None	1+	Normal	Normal	Normal	Reduced
	EDC, EIP	None	None	Normal	Normal	Normal	Reduced
	Lower cervical PSP	None	None				
Case 3	APB	None	None	Normal	Normal	Normal	Complete
	FCU	2+	None	Normal	↑	Normal	Complete
	ADM	1+	None	Normal	↑	Normal	Reduced
	FDI	3+	None	Normal	↑	Normal	Reduced
	Lower cervical PSP	None	None				

ASA: abnormal spontaneous activity; MUAP: motor unit action potential; R: recruitment; PSWs: positive sharp waves; Fibs: fibrillation potentials; Amp: amplitude; Dur: duration; IP: interference pattern; APB: abductor pollicis brevis; ADM: abductor digiti minimi; EIP: extensor indicis proprius; FCU: flexor carpi ulnaris; FDI: first dorsal interosseus; EDC: extensor digitorum communis; EPL: extensor pollicis longus; PSP: paraspinalis; ↑: increased.

## Data Availability

Data are available when on request.
